# Low-level mediation of directionally specific motion aftereffects: Motion perception is not necessary

**DOI:** 10.3758/s13414-016-1160-1

**Published:** 2016-07-08

**Authors:** M. J. Morgan, K. Schreiber, J. A. Solomon

**Affiliations:** 1 Max-Planck Institute for Metabolism, Cologne, Germany; 2 City University London, London, UK

**Keywords:** Adaptation, Aftereffects, Attention, 2-D Motion

## Abstract

Previous psychophysical experiments with normal human observers have shown that adaptation to a moving dot stream causes directionally specific repulsion in the perceived angle of a subsequently viewed moving probe. In this study, we used a two-alternative forced choice task with roving pedestals to determine the conditions that are necessary and sufficient for producing directionally specific repulsion with compound adaptors, each of which contains two oppositely moving, differently colored component streams. Experiment 1 provided a demonstration of repulsion between single-component adaptors and probes moving at approximately 90° or 270°. In Experiment 2, oppositely moving dots in the adaptor were paired to preclude the appearance of motion. Nonetheless, repulsion remained strong when the angle between each probe stream and one component was approximately 30°. In Experiment 3, adapting dot pairs were kept stationary during their limited lifetimes. Their orientation content alone proved insufficient for producing repulsion. In Experiments 4–6, the angle between the probe and both adapting components was approximately 90° or 270°. Directional repulsion was found when observers were asked to visually track one of the adapting components (Exp. 6), but not when they were asked to attentionally track it (Exp. 5), nor while they passively viewed the adaptor (Exp. 4). Our results are consistent with a low-level mechanism for motion adaptation. This mechanism is not selective for stimulus color and is not susceptible to attentional modulation. The most likely cortical locus of adaptation is area V1.

**Electronic supplementary material:**

The online version of this article (doi:10.3758/s13414-016-1160-1) contains supplementary material, which is available to authorized users.

Psychophysical and physiological evidence combine in suggesting that motion processing within the central visual system occurs in at least two stages (Movshon & Newsome, 1996). In the first stage, motion signals are measured within local regions of visual space by mechanisms whose preferred directions are orthogonal to their preferred axes of orientation, but that nonetheless respond to all directions within ±90° of their preference, due to the “aperture problem.” Veridical estimates of direction can be obtained when multiple first-stage signals are combined using the “intersection of constraints” rule (Adelson & Movshon, 1982; Ferrera & Wilson, 1990; Movshon, Adelson, Gizzi, & Newsome, 1985; Rodman & Albright, 1989).

Evidence for the two-stage model comes from experiments on transparent motion. When two sets of independently positioned dots move in opposite directions, both directions of motion are visible. Snowden, Treue, Erickson, and Andersen (1991) showed that V1 neurons stimulated by one direction of moving dots were largely unaffected when dots moving transparently in the opposite direction were added to the stimulus. Most neurons sampled from MT, on the other hand, show some degree of suppression from dots moving the opposite direction (unless they are given a binocular disparity, which makes them appear in a different depth plane; Bradley, Qian, & Andersen, 1995). This finding suggests that motion signals are averaged over a larger spatial scale in MT, possibly for the purposes of noise reduction and smoothing (Qian & Andersen, 1994).

Qian and Andersen (1994) replicated these findings, using oppositely moving dots that were paired in close spatial proximity. V1 neurons were affected little by the pairing, whereas MT neurons tended to be suppressed. Qian, Andersen, and Adelson (1994) had previously noted that neither direction of motion was seen in the paired-dot display. The display only seemed to flicker.

Analogous results have been obtained with drifting gratings. They activate individual neurons (Qian & Andersen, 1994) and produce positive blood oxygenation level dependent (BOLD) responses (Heeger, Boynton, Demb, Seidemann, & Newsome, 1999) in both V1 and MT, but whereas the addition of otherwise identical, oppositely drifting gratings suppresses the responses in MT, it does not suppress the response in individual neurons or the magnitude of the BOLD response in V1. Apparent motion is also absent from this “counterphasing” stimulus. It too merely appears to flicker.

Some of the best evidence for the two-stage model has come from adaptation experiments. For example, Kohn and Movshon (2003) showed that adaptation to small patches of drifting grating could reduce the contrast gain of directionally selective MT neurons in anaesthetized, paralyzed macaque monkeys. However, this happened only when the adapting and probe stimuli were presented in the same, small subarea of the MT neuron’s receptive field. Kohn and Movshon inferred from this result that the primary locus of adaptation is in the smaller receptive fields of V1 neurons, and that this adaptation is merely inherited by MT. We can conjecture that MT neurons would similarly inherit adaptation from V1 when the latter was stimulated with counterphasing gratings or the paired-dot stimulus.

There have been many psychophysical demonstrations of adaptation to moving stimuli. Prolonged inspection of a drifting grating or drifting dots is known to produce a selective loss of sensitivity to movement in the adapting direction (Morgan, Chubb, & Solomon, 2011; Sekuler & Ganz, 1963), a reduction of the perceived velocity in the adapting direction (Thompson, 1981), and repulsion of the perceived angle of motion away from the adapting angle (Levinson & Sekuler, 1976). In this study, we examined motion adaptation to paired dots. The two-stage model of motion perception predicts that adaptation to paired-motion stimuli or counterphasing gratings should result in selective adaptation to both directions of motion. Consistent with this prediction, we report repulsion of the perceived angle of motion away from both angles in the adapting stimulus.

Our study is a straightforward extension of Levinson and Sekuler’s (1976). They used transparently moving (i.e., unpaired) dots. Human observers were adapted to a set of dots moving at 120° (i.e., up and to the left), combined with a set moving at 300°. We shall use the notation *120/300* for this stimulus. Following adaptation, observers were shown probes at 90° and adjusted the orientation of a line to their perceived direction of movement. The probe was repelled away from the 120° component of the adapting stimulus by the same amount as it had been from an adaptor containing a single 120° component. (We refer to this as *120/120*.) However, no repulsion of a 90° probe occurred from a 300/300 adaptor.

We predicted a similar result with adaptation to a paired-dot moving stimulus, even though it is seen as flickering rather than moving. To test the prediction, we adapted to a 30/210 paired-dot stimulus and tested with probe dot streams moving at 0° and 180°. We predicted that both probes would show clockwise (CW) repulsion. To measure the effect, we analyzed the psychometric functions from a two-alternative forced choice (2AFC) task with roving pedestals. This allowed us to determine the actual angle at which the probes appeared to the observer to move horizontally. To show that the predicted CW shift was not a static tilt aftereffect, we used a control in which the paired dots formed a Glass pattern, with clear orientation but no movement.

The only previous study of adaptation to paired motion of which we are aware was performed by Blaser, Papathomas, and Vidnyanszky (2005), who used the same logic as ours to predict repulsion of orientation from the components. These authors adapted to 0/180 and tested at 90°. No repulsion would be expected in this case, when the two sets of dots have the same motion energy, because the probe would be repelled in opposite directions by the two components. However, Blaser et al. used different colors for the leftward- and rightward-moving dots, and reported repulsion of red probes from red adaptors, and green from green. In other words, the effects of adaptation were color-specific. To test for color specificity using our own 2AFC psychophysical methods, we adapted to red 0° and green 180° (R0/G180) and tested with R0, R180, G0, and G180 probes.

## General method

Stimuli were presented on a 60-Hz frame-rate Sony Trinitron monitor, viewed from 75 cm, so that one pixel subtended 1.275 arcmin at the observer’s eye. Except where otherwise stated, the viewing parameters were as close as possible to those of Blaser et al. (2005). The circular aperture size was 4.25°, the dot diameter was 0.0425°, the dot lifetime was five frames (80 ms), and the velocity of the adapting dot movement was 2.5°/s. The number of dots was 256 (or 128 green and 128 red, in the transparent condition). The initial adaptation period was 40 s, and subsequent “top-up” periods were 8 s each. The background screen luminance was 50 cd/m^2^ in Experiment 1, but ~0 cd/m^2^ in Experiments 2–6, as in the experiments reported by Blaser et al. The central fixation point was a 0.05° white square. (Blaser et al. also had a central fixation point, but its size was not specified.)

The luminances of the red and green dots were chosen to be equally salient in the transparent stimulus. Blaser et al. (2005) did not specify their dot luminance values, but stated that they were calibrated for isoluminance for each participant. (Presumably isoluminant with each other, not with the dark background.) Except in experiments with transparent motion, we used only green dots.

Eye position was measured with an EyeLink 1000 far-infrared reflection recorder.

**Fig. 1:**
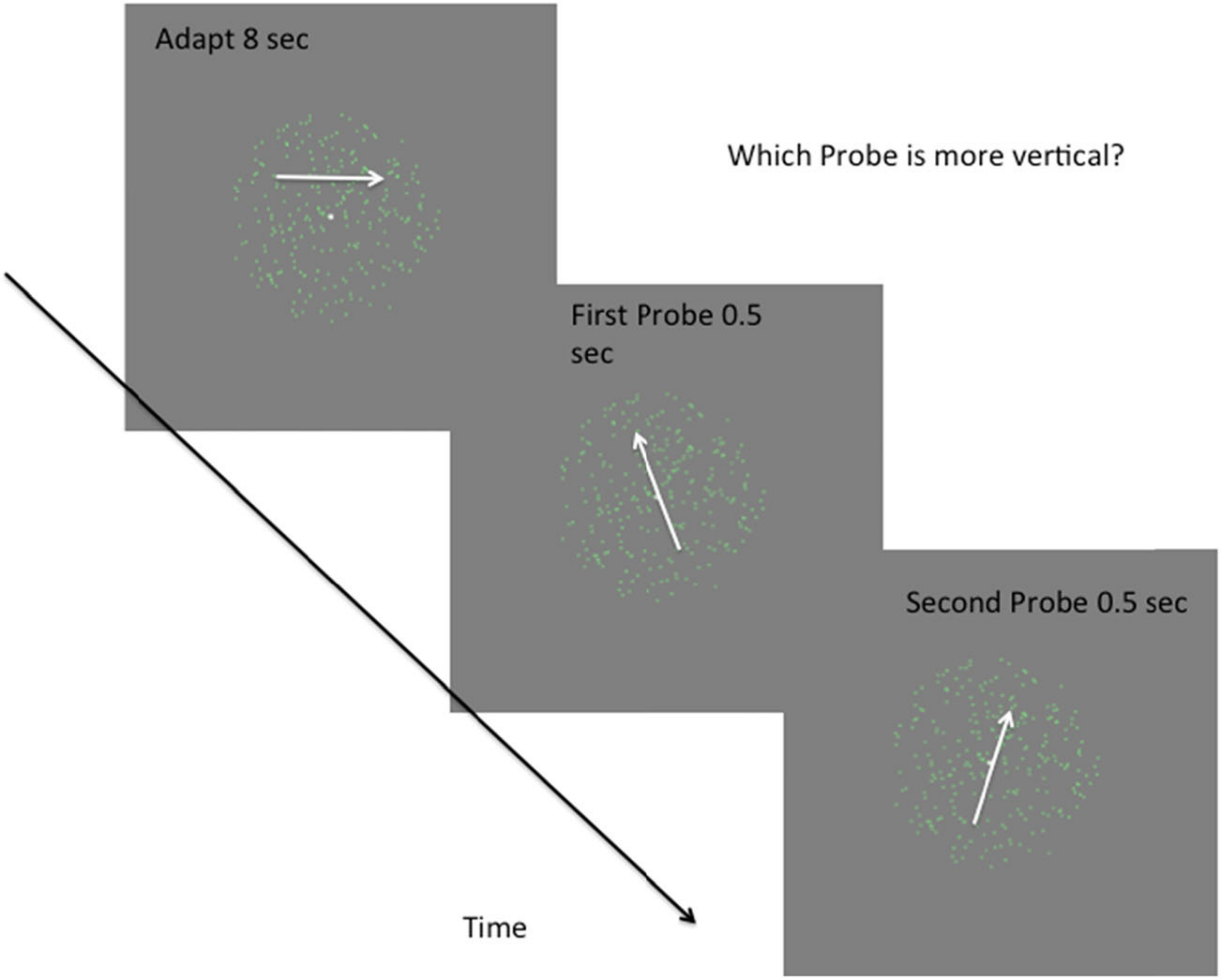
Schema of the experimental procedure. In experiments with motion transparency, the adapting stimulus was replaced by equal numbers of red and green dots, moving in opposite directions

The stimuli and a typical trial sequence are illustrated in Fig. 1. (See also the file DemoAdaptRedTestRed.mp4 in the supplementary material.) Each session began with a 40-s adaptation period, during which the observer was instructed to maintain fixation. This was followed by a sequence of 192 trials. Every 50 trials, the observer was instructed by a message on the screen to take a rest, following which a keypress initiated another 40-s adaptation period. On all other trials, the adaptation period was 8 s. The adapting stimulus consisted of 256 green dots randomly scattered in the circular aperture. Each of these dots moved rightward with a limited lifetime of five frames (Morgan & Ward, 1980a, b), at the end of which it was replaced by a dot in a random position within the aperture. Any dot that reached the edge of the aperture was wrapped to the mirror image position on the aperture, with a small horizontal shift toward the center equal to two dot diameters.

Our psychophysical method combined 2AFC with a roving pedestal (Morgan, Melmoth, & Solomon, 2013). This combination was designed to obscure the relationship between our hypotheses and the observer’s response. This was advantageous because it prevented simple cognitive biases from masquerading as a true perceptual bias (cf. Morgan, Dillenburger, Raphael, & Solomon, 2012).

Each “adaptor” was followed by two probe stimuli. A 0.2-s delay preceded each 0.5-s probe. Although the two probes moved in slightly different directions (see below), both directions were close to the “reference” direction, which could be either straight up, straight down, left, or right. The observer’s task was to press a key (“1” or “2”) to indicate which of the two probes appeared to move in a direction closest to the reference direction. We refer to one probe as the *pedestal*. Its direction of motion was selected from the pedestal angles *p* ∈ {–10°, 0°, 10°} with respect to the reference. The other probe moved in a direction that was the sum of this same pedestal and a “test level,” randomly selected from the set *t* ∈ {–16°, –12°, –8°, –4°, 4°, 8°, 12°, 16°}. We refer to this probe as the *test* stimulus. Note that the angles of the two probes could be on opposite sides of the reference. Each of the 8 × 3 × 2 kinds of trials was repeated in a random sequence without replacement, making a total of 192 trials per session.

The data from each session were fit with a two-parameter signal-detection model, to obtain values of the observer’s bias (*μ*) and the just-noticeable difference (JND; *σ*). These correspond intuitively (but not mathematically) to the 50% point and the inverse slope of the psychometric function in the method of single stimuli (MSS), as used for example by Blaser et al. (2005).

### Signal-detection model

Within the context of signal-detection theory (Green & Swets, 1966), the apparent directions of the two probes can be described by normal distributions *S* and *T*, such that *S* ~ N(*p* + *μ*, *σ* ^2^/2) and *T* ~ N(*p* + *t* + *μ*, *σ* ^2^/2), where *σ* ^2^ is the variance of the performance-limiting noise, *p* and *p* + *t* represent the physical directions of drift, and *μ* represents any perceptual bias, such as may be induced by adaptation. Given these definitions, the probability of choosing the pedestal is given by

1 $$ \begin{array}{c}\hfill \Pr \left(^{{\prime\prime}}\mathrm{S}^{{\prime\prime}}\right)= \Pr \left(\left|S\right|<\left|T\right|\right)\hfill \\ {}\hfill = \Pr \left(\frac{S^2}{T^2}<1\right).\hfill \end{array} $$

Morgan et al. (2015) noted that *S* ^2^/*T* ^2^ is a random variable having a doubly noncentral *F* distribution. Its denominator’s noncentrality parameter is 2(*p* + *t* + *μ*)^2^/*σ* ^2^, its numerator’s noncentrality parameter is 2(*p* + *μ*)^2^/*σ* ^2^, and both denominator and numerator have one degree of freedom. However, evaluating the doubly noncentral *F* distribution can be computationally intensive. Here we provide an equivalent formulation, which can be calculated very quickly:

2 $$ \begin{array}{c}\hfill \Pr \left(^{{\prime\prime}}\mathrm{S}^{{\prime\prime}}\right)= \Pr \left(\frac{S^2}{T^2}<1\right)\hfill \\ {}\hfill =\left(1+\mathrm{e}\mathrm{r}\mathrm{f}\left[t/\left(2\sigma \right)\right]\mathrm{e}\mathrm{r}\mathrm{f}\left[\left(2\mu +2p+t\right)/\left(2\sigma \right)\right]\right)/2.\hfill \end{array} $$

The participants were the three authors (M.M., J.S., and K.S.), four psychophysically experienced colleagues (B.D., J.F., A.J., and N.N.) not involved in the design of the experiment, and two paid volunteer undergraduates (T.P. and D.P.) who were not aware of the purpose of the experiment. Not all participants took part in all experiments.

## Experiment 1

The purpose of the first experiment was to measure the size of the orientation repulsion effect using our own methods and stimuli, and to introduce the reader to the analyses used in the subsequent experiments. Observers adapted to a single component moving at 0° (horizontally to the right), and were tested with both upwardly and downwardly moving probes, randomly interleaved within a single session (sampling without replacement). On each trial, after a top-up adaptation, two stimuli were presented in temporal succession, and the observer had to report which of them was closer to the vertical. (See the General Method section.)

### Results

**Fig. 2:**
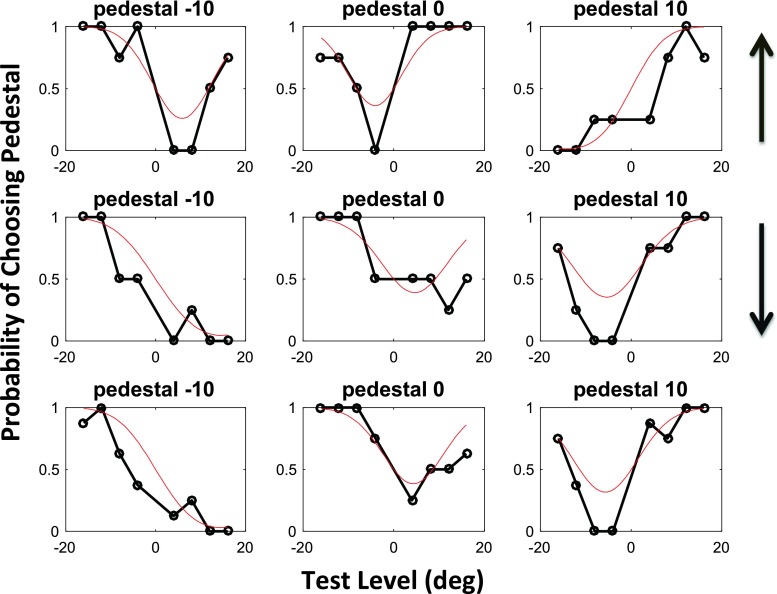
Psychometric functions obtained from one observer (T.P.) in Experiment 1. The arrows show the movement directions of the reference. The bottom row shows the data for the top two rows combined, with reversal of the pedestal and test levels in the top condition. For further explanations, see the text. Note that the test levels (horizontal axis) are added to the pedestal value in the test stimulus. Positive values are counterclockwise

Examples of the raw psychometric functions from which we derived estimates of bias and the JND are shown in Fig. 2. These were derived from a single testing session with one naive observer (T.P.) comprising 192 trials (3 pedestals × 8 test levels × 2 reference directions × 4 repeats). The first row shows the results for one reference direction (90°; see the arrow to the right), and the second row shows those for the other reference direction (270°). The vertical axis shows the probability that the observer chose the pedestal, rather than the test (horizontal axis). The solid symbols show the data, each point being based on only four repeats, which explains the quantization of the probabilities to only five levels. The third row shows the data from the first two rows combined, with a reversal of the test and pedestal values of the first row, to take account of the reverse biases expected for the 90° and 270° cases.

The data in Fig. 2 are best summarized within the context of signal-detection theory. Nonetheless, a rough estimate for the size of the motion aftereffect can be obtained from inspecting the raw psychometric functions. First consider those obtained with pedestals of zero. With a zero pedestal and a zero test level, we expected the observers to choose the pedestal 50% of the time, even if they had a perceptual bias. Furthermore, if the rightward-moving adaptor produced counterclockwise (CCW) biases (i.e., positive angles) in the observer’s percepts of both probe stimuli, then the observer should be less likely to choose any particular probe (as being more vertical) when an additional CCW angle was added to it. The results in the top row (central panel) are consistent with this prediction. Observer T.P. invariably selected the pedestal as being more vertical whenever a CCW angle was added to the test. Conversely, probes containing a CW (negative) test level might appear closer to vertical, making observers less likely to select the pedestal. The observer should be least likely to select the pedestal when the cue level is exactly opposite the observer’s bias, and the psychometric function should be symmetric around this value.

Now consider the case in which there is a nonzero pedestal. If the pedestal was in the same direction as the observer’s bias, both probes would seem shifted from the vertical by amounts equal to the bias and the pedestal. Test levels in one direction would make the test look more vertical than the pedestal, test levels in the other direction would make it look less vertical. Consequently, the psychometric function should be sigmoidal in the region around the point (0, .5). See the top right and middle left panels for examples.

Finally, consider the case in which the pedestal and bias are in opposite directions. In this case, a small test value (positive or negative) would make the motion of the test indiscriminably different from vertical, and consequently the observer should only rarely select the pedestal. Results of this nature can be seen in the top left and middle right panels.

Inspection of the raw data in Fig. 2 makes clear that adaptation to rightward motion produced a positive (CCW) bias in the perception of upward-moving probes (top row of panels) and a negative (CW) bias in the perception of downward-moving probes (middle row). Biased functions like these can be compared to the unbiased functions obtained from the “non-frame-dependent” participants in a rod-and-frame task (see Morgan et al., 2015, Fig. 3).

**Fig. 3:**
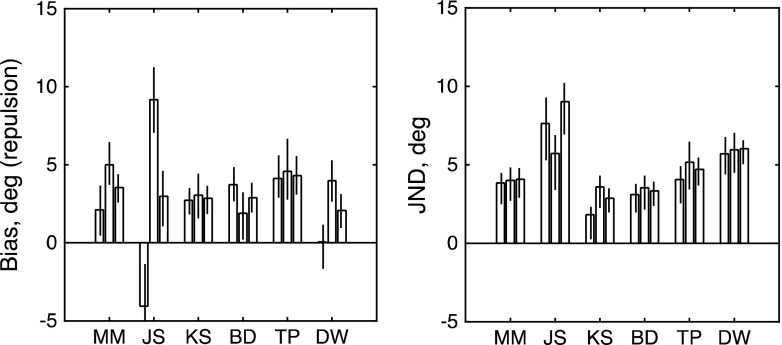
Results of Experiment 1. The left-hand and right-hand panels show maximum-likelihood estimates of bias (*μ*) and JND (*σ*) for each observer. From left to right, the three bars for each observer show estimates derived from (1) trials with an upward reference, (2) trials with a downward reference, and (3) all trials fit together. Each error bar contains the central 95 percentiles of a parametric bootstrap distribution (sample size: 1,600)

The red curves in Fig. 2 show the fits of the signal-detection model. This two-parameter model was simultaneously fit to all 96 trials depicted in the top row; it was fit again to all 96 trials depicted in the middle row; and finally it was fit to all 192 trials in the bottom row. The results of these fits are summarized in Fig. 3. The sign of the biases is in the direction expected if the probes were repulsed from the 0° adaptor. Thus, upward-moving dots are apparently displaced CCW (positive bias), and downward-moving probes are displaced CW (negative bias). The rightmost bar for each observer shows the net repulsion effect, obtained by combining the same direction of test. This is positive in all observers. One observer (J.S.) had a large overall CW bias, which inverted the repulsion to an apparent attraction to the upward reference, but his combined data were in the repulsion direction. The values for bias (left-hand panel) and JND (right-hand panel) are quite similar, as is commonly found when applying the MSS to the measurement of classical perceptual biases such as the Müller-Lyer (Morgan, Hole, & Glennerster, 1990) and in 2AFC measures of the “rod-and-frame” effect (Melmoth, Grant, Solomon, & Morgan, 2015). To test whether the biases were significantly different from zero, we used a log-likelihood analysis, comparing the two-parameter fit (*μ*, *σ*) to a constrained fit with *μ* set to zero. Under the null hypothesis (i.e., *μ* = 0), twice the difference in log likelihoods between the two fits is distributed as *χ* ^2^ with *df* = 1 (Hoel, Port, & Stone, 1971). The values of this test statistic for the six observers (in the order shown in Fig. 3) were 23.7872, 5.3444, 19.5877, 20.6917, 28.5069, and 8.0290. All these values are larger than that (5.024) required to reject the null hypothesis at the *α* = .025 level of significance.

These results confirm the report by Levinson and Sekuler (1976) that a moving dot stream is repulsed away from the direction of an orthogonal adapting stream.

## Experiment 2

Having confirmed the repulsion effect of Levinson and Sekuler (1976) with our own method, we used it to determine whether there is adaptation to paired motion (Qian et al., 1994). Six observers were tested with adaptation to 30/210 (i.e., oblique) adaptors. Two of these six (M.M. and K.S.) were, in addition, adapted to 150/330. (See the General Method section.) The results for 30/210 were combined with those for 150/330, after reversal of the test and pedestal values for the latter, so that a positive bias would represent repulsion. Trials with leftward and rightward references were randomly interleaved. Data were analyzed in the same way as in Experiment 1.

### Results

**Fig. 4:**
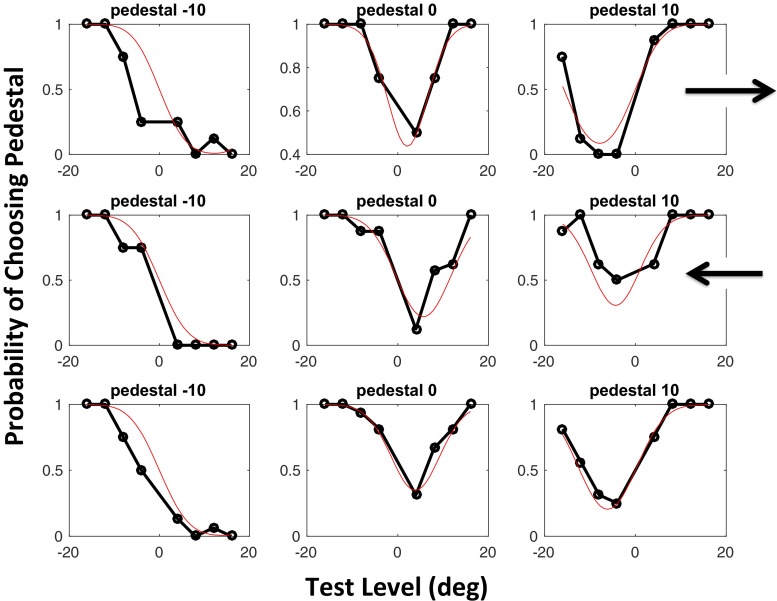
Psychometric functions obtained from one observer (M.M.) in Experiment 2, based on a total of 381 trials. The arrows show the reference directions, and the bottom row shows the data for the top two rows combined. For further explanations, see the text

**Fig. 5:**
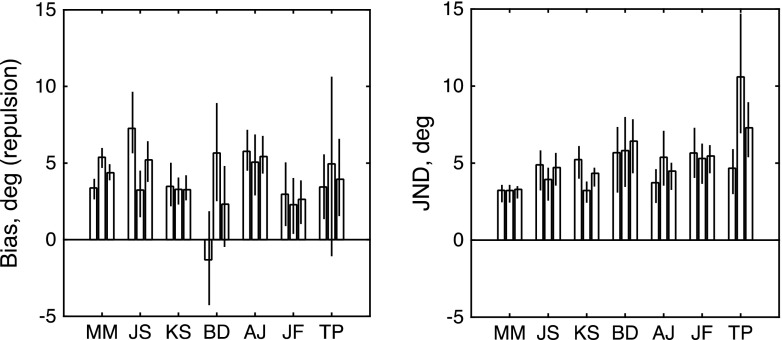
Results of Experiment 2, in which the adapting stimulus consisted of paired dots moving in opposite directions. The left-hand and right-hand panels show maximum-likelihood estimates of bias (*μ*) and JND (*σ*) for each observer. From left to right, the three bars for each observer show estimates derived from (1) trials with a rightward reference, (2) trials with a leftward reference, and (3) all trials fit together. Each error bar contains the central 95 percentiles of a parametric bootstrap distribution (sample size: 1,600). As in Fig. 3, the *μ* values are expressed as the angles of repulsion

The psychometric functions for one observer (M.M.) are shown in Fig. 4. In this case, unlike in Fig. 2, we find the same direction of bias for both reference directions, so the third row shows the results for the first two rows combined, without reversal of sign. Summary results are shown in Fig. 5. All observers show a net bias (rightmost bar in each group) in the predicted direction, although B.D. has a strong CCW bias that destroys the symmetry of her data. The test statistics for our log-likelihood analysis were 127.2109, 35.9124, 32.8900, 2.3710, 40.2409, 10.3021, and 6.9878, respectively, for the observers shown in Fig. 5. Thus we can reject the null hypothesis (*μ* = 0) for six of our seven observers. A *t* test of the net biases showed that they were significantly different from a distribution of observers with zero mean: *t*(6) = 8.47, *p* = .00015.

## Experiment 3

Although the results of the previous experiment may seem compelling evidence for directionally specific adaptation, an alternative interpretation is based on the static tilt aftereffect (Gibson & Radner, 1937; Meese & Georgeson 1996). Indeed, the paired-dot stimulus had a strongly striated appearance, along the axis of motion. These “motion streaks” could have affected the apparent orientation of similar streaks in the probe stimuli, and the latter could have affected judgments of motion direction (Geisler, 1999).

Levinson and Sekuler (1976) discussed this objection to their interpretation of transparent motion adaptation, and rejected it on the cogent grounds that adaptation to a single component direction is directionally specific. For example, adaptation to 120/300 produces CW repulsion of a 90° probe, as does adaptation to 120/120: but adaptation to 300/300 produces no repulsion with such a probe. If adaptation were based on motion streaks, then 120° and 300° adaptors should have the same effect, since they differ only in direction, not in orientation.

To satisfy ourselves on this point, we replicated Levinson and Sekuler’s experiment with three observers (M.M., A.J., J.S.), and obtained the same results (not shown here). However, this rebuttal of streaks is not completely convincing for the case of paired dots, because it is possible that streaks would be stronger in this case than for a single direction of moving dots. We therefore designed a stimulus that had a strongly oriented structure but no motion. This consisted of the paired dots used in the previous experiment, but they did not move during their lifetime. Observers B.D., A.J., and J.F. were adapted to 30/210. Observer J.S. was adapted to 150/330. Observers M.M. and K.S. experienced both conditions in different sessions. The results for 30/210 were combined with those for 150/330, after reversal of the cue and pedestal values for the latter, so that the overall bias would represent a repulsion. The stimulus had a strongly striated appearance, as would be expected from a Glass (1969) pattern, but had no motion along the axis of the striations. Such motion as there was in the pattern was orthogonal to the striations, arising from the nonuniform distribution of motion energy imposed by the orientation structure (cf. Morgan & Tyler, 1995, who used a cylindrical lens to study the Pulfrich effect with random dynamic noise).

### Results

**Fig. 6:**
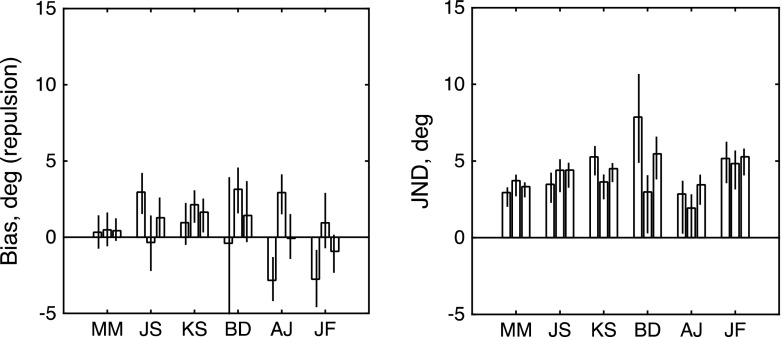
Results of Experiment 3, in which the adapting stimulus consisted of stationary paired dots. The left-hand and right-hand panels show maximum-likelihood estimates of bias (*μ*) and JND (*σ*) for each observer. From left to right, the three bars for each observer show estimates derived from (1) trials with a rightward reference, (2) trials with a leftward reference, and (3) all trials fit together. Each error bar contains the central 95 percentiles of a parametric bootstrap distribution (sample size: 1,600)

The summary results are shown in Fig. 6. For only one of the six observers (K.S., who had a strong overall CW bias) was the net bias significantly different from zero. (The values of the test statistic for the log-likelihood analysis were 0.9485, 3.2081, 8.5696, 1.2002, 0.0056, and 0.9527, respectively, for the observers shown in Fig. 6.) A group *t* test showed that the difference from zero was not significant: *t*(5) = 1.582, *p* = .1745. This result contrasted with the paired-motion case [Exp. 2: t(6) = 8.45, *p* = .0015]. Another paired *t* test showed that the difference between the two experiments in those observers who did both was also significant: *t*(5) = 4.644, *p* = .0056. We conclude that the adaptation found with moving paired dots is unlikely to be explained by the static tilt aftereffect.

## Experiment 4

Blaser et al. (2005) described directionally specific repulsion of a 90° probe, following adaptation to both a transparent and a paired-dot stimulus with 0/180 components. This adaptation was unexpected, because the two components should cancel out. However, the two sets of moving dots were colored red and green, and the adaptation was found to be color-specific. We tried to repeat this result using our own stimuli and psychophysical methods. We adapted to a 0/180 transparent stimulus of rightward-moving green dots (0°) and leftward-moving red dots (180°). Next we tested with interleaved upward (90°) and downward (270°) references, exactly as in Experiment 1. (For a demo, see supplementary file DemoAdaptTransTestRedandGreen.mp4.) In separate sessions, the probe dots were either red or green. If a color-contingent motion adaptation effect was present from a transparent stimulus, we would find opposite directions of repulsion with the two different probe colors.

**Fig. 7:**
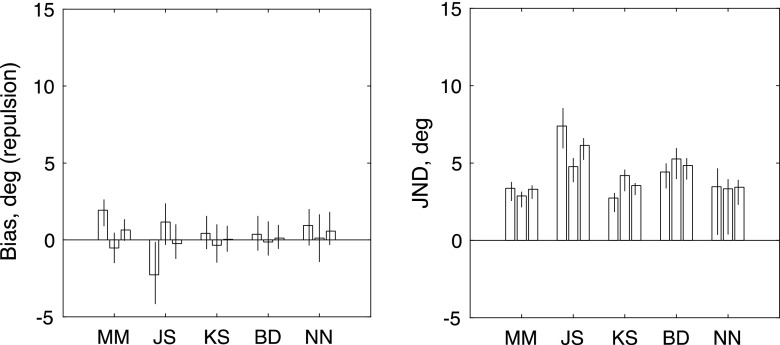
Results of Experiment 4. The left-hand and right-hand panels show maximum-likelihood estimates of bias (*μ*) and JND (*σ*) for each observer. From left to right, the three bars for each observer show estimates derived from (1) trials with an upward reference, (2) trials with a downward reference, and (3) all trials fit together. Trials with green probes and red probes have been combined. Each error bar contains the central 95 percentiles of a parametric bootstrap distribution (sample size: 1,600)

Figure 7 shows three bars for each observer. From left to right, the three bars show estimates derived from (1) trials with an upward reference, (2) trials with a downward reference, and (3) all trials fit together. The results for the two colors are combined with an appropriate sign reversal, so that a positive effect would indicate repulsion. Clearly there was no significant net bias. The values of the test statistics for the log-likelihood analysis were 2.2334, 0.1068, 0.0061, 0.0567, and 0.9399, respectively, for the observers shown in Fig. 7. Thus we cannot reject the null hypothesis (*μ* = 0) for any of our five observers.

We concluded that our psychophysical technique does not produce any evidence for significant color-specific, directionally selective motion adaptation from a transparent stimulus.

## Experiment 5

We wondered whether Blaser et al. (2005) obtained color-contingent adaptation by involuntarily attending to one of the components in the adapting stimulus. After attending to red, for example, there might be an adaptation specific to the movement direction of the adapting red dots. This would be a direction-specific adaptation, not a color-specific effect. Just such an effect has been reported (Lankheet & Verstraten, 1995), albeit it with a different stimulus array and a different psychophysical procedure. (They used MSS to find the null point in the signal-to-noise ratio.) To examine this possibility, we repeated Experiment 5, but with attention to one component of the transparent stimulus. Observers attempted to follow the motion of either the green or the red dots “in the mind’s eye,” but without actually tracking them. We admit that these instructions were not very precise, and could elicit a number of different strategies, such as attempting to follow individual dots attentively or attending to a particular apparent depth plane. We verified informally with the EyeLink recorder that observers were not tracking the targets. In ATTEND TO RED blocks, the probe stimuli were red. In ATTEND TO GREEN blocks, they were green. Thus, a possible direction-specific adaptation was confounded with a possible color-contingent adaptation, as in the Blaser et al. experiment. (Though not, we think, in Lankheet & Verstraten, 1995, where the color of the probes was not the same as that of the attended component.)

**Fig. 8:**
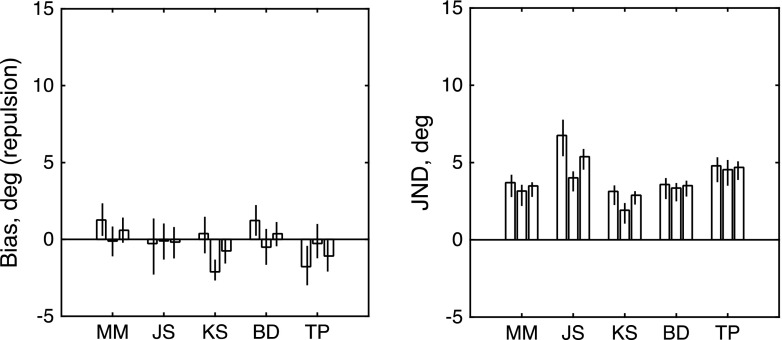
Results of Experiment 5. The left-hand and right-hand panels show maximum-likelihood estimates of bias (*μ*) and JND (*σ*) for each observer. From left to right, the three bars for each observer show estimates derived from (1) trials with a rightward reference, (2) trials with a leftward reference, and (3) all trials fit together. Trials with attend-to-green and attend-to-red probes have been combined. Each error bar contains the central 95 percentiles of a parametric bootstrap distribution (sample size: 1,600)

The results (Fig. 8) showed no significant net effect of the attended color on adaptation. The values of the test statistic for the log-likelihood analysis were 1.5563, 0.0711, 2.9851, 0.6382, 3.5382, and 3.841, respectively, for the five observers (M.M., J.S., K.S., B.D., T.P.). Thus, we cannot reject the null hypothesis (*μ* = 0) for any of our five observers.

## Experiment 6

A possible explanation of adaptation to transparent motion is pursuit eyetracking (see the Discussion section). To test the possible role of tracking, we adapted observers to a transparently moving stimulus while they were instructed to pursue a white fixation point moving with the same velocity as one of the components. The actual movement of the fixation point had a saw-tooth shape, and it moved instantaneously to the left-hand side of the circular aperture (Fig. 1) when it reached the right-hand edge.

**Fig. 9:**
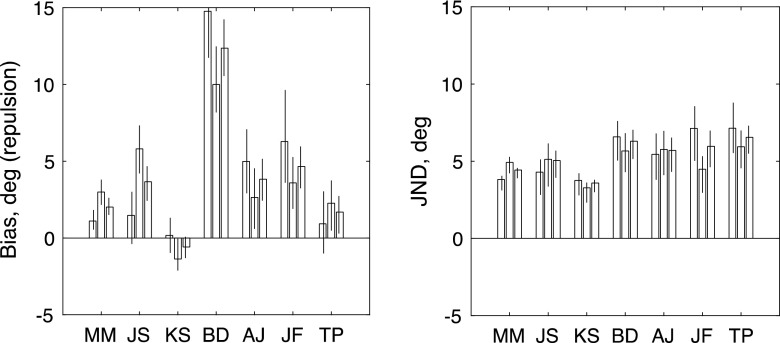
Results of Experiment 6. The left-hand and right-hand panels show maximum-likelihood estimates of bias (*μ*) and JND (*σ*) for each observer. From left to right, the three bars for each observer show estimates derived from (1) trials with an upward reference, (2) trials with a downward reference, and (3) all trials fit together. Each error bar contains the central 95 percentiles of a parametric bootstrap distribution (sample size: 1,600)

Figure 9 shows the results for observers M.M., J.S., K.S., B.D., A.J., J.F., and T.P. All observers showed an aftereffect in the expected direction (repulsion from the direction of tracking). Their values of the test statistic in the log-likelihood analysis were 31.7869, 16.6479, 1.4562, 106.4826, 16.3963, 20.7043, and 4.5260, respectively. Thus we can reject the null hypothesis (*μ* = 0) for six of our observers, but not for K.S. Overall, despite the high variance between observers, the data allowed us to reject the null hypothesis that the seven observers were drawn from a population with mean of zero [*t*(6) = 2.55, *p* = .0437].

## Discussion

The results of our first experiment (Exp. 1) confirmed the finding by Levinson and Sekuler (1976) that a horizontal moving adaptor causes repulsion in orthogonal probes (0° and 270°). The results of our Experiment 2 supported the claim by Blaser et al. (2005) that motion adaptation can be produced by a paired-dot stimulus (Qian et al., 1994). We found that a 30/210 paired-dot adaptor caused directional repulsion in both 0° and 180° moving probes. The finding of adaptation to paired motion, added to the further finding by Levinson and Sekuler that adaptation to one component of a transparently moving stimulus is no weaker than to a single component, gives strong psychophysical support to the two-stage model of motion processing (Adelson & Movshon, 1982; Movshon & Newsome, 1996). According to the two-stage model, elaborated to include adaptation, V1 neurons respond to one component of paired-dot or transparently moving stimuli as if the other component were absent. V1 neurons also adapt to their input (Kohn & Movshon, 2003), and these two facts taken together imply that they would adapt to both paired-dot and transparent stimuli, as we and Levinson and Sekuler both found. MT neurons, on the other hand, merely inherit their adaptation from V1 and combine, to a greater or lesser extent, motion in opposite directions within their receptive field. This is generally held to explain why paired-dot stimuli are not seen to move, although the linking hypothesis here has not been made clear or justified. Presumably the hypothesis is that perception should be linked more to later than to earlier stages in a processing hierarchy, because later stages are closer to the response buttons or tongue.

On the other hand, our results (Exp. 4) did not confirm the factual basis for the claim (Blaser et al., 2005) that a 90° probe is repulsed from both components of a 0/180 paired-dot adaptor. Such repulsion would not be expected according to our logic, since the two adapting components would cancel out. Blaser et al. attempted to prevent this cancellation by making the oppositely moving dots of different colors and testing with single colors. Since our experiment was a conceptual (Schmidt, 2009) rather than an exact replication, we cannot be certain why our results are different. Differences include the psychophysical method (2AFC rather than MSS, which has one stimulus and two possible responses), the statistical methods of analysis, the use of colors that appeared equally salient to the observer, rather than equiluminous, and the absence in our experiment of stationary dots in the opposite color from the moving probe, which were present in Blaser et al.

Differences in the outcomes of different psychophysical procedures have already been noted elsewhere, and perhaps deserve more attention. Mather and Sharman (2015) have argued that the claim for adaptation based on imagining the adaptor (Winawer, Huk, & Boroditsky, 2010) depends on response bias with the MSS. When the decision was changed from “which direction is the probe moving” to “in which half of the stimulus array is there coherent movement,” the effect of an imaginary adaptor disappeared. Similarly, using a 2AFC procedure, Morgan (2014) failed to find spatiotopic adaptation of tilt adaptation, which had been reported by Turi and Burr (2012) using the MSS. In another example, again using 2AFC, Morgan (2011, 2013) failed to find an effect of attentional load during motion adaptation, which had been reported by Taya, Adams, Graf, and Lavie (2009) using the MSS. On the other hand, there are good reasons for rejecting response bias as an explanation for the paired-motion findings of Blaser et al. (2005), since they showed that participants were unable to report the association between color and motion in a forced choice task.

Concerning statistical procedures, we have little to say. Blaser et al. (2005) presented only group data in their article. Individual psychometric functions were not analyzed, and the significant result applied to the group data (Blaser, personal communication). It is possible, therefore, that some observers, including those that were naive, did not show a significant effect. This is an important difference from our analysis, in which we considered the observers separately, except where we report population *t* tests.

Although our manipulation of attention did not produce a directional aftereffect, Lankheet and Verstraten’s (1995) manipulation of attention did. The reason for this discrepancy remains unclear. One possibility is that our observers used a less effective strategy for maintaining one component “in the mind’s eye.” Another obvious difference is that we used a directional repulsion effect, whereas Lankheet and Verstraten measured the dynamic-motion aftereffect with a signal-to-noise ratio method.

We tried informally to find a dynamic-motion aftereffect after attending to transparent red–green motion, by using probes composed of stationary dots. (Each dot had a limited lifetime of five frames.) This produced a clear motion aftereffect after adaptation to a single direction (red dots only; see DemoAdaptRedTestDVN.mp4), but all we could see after transparent adaptation (DemoAdaptTransTestDVN.mp4), with or without selective attention, was the vague motion orthogonal to the axis of adaptation predicted (and found) by Grunewald and Lankheet (1996). The generality of attention-contingent adaptation clearly needs further investigation. Raphael, Dillenburger, and Morgan (2010) examined the effect using transparent streams of expanding/contracting black/white dot streams. They did find an effect, but it was noisy and inconsistent over observers. The main effect was a massive, idiosyncratic bias toward reporting either “expanding” or “contracting.”

Another possible mechanism for the aftereffect of transparent motion is pursuit tracking of one of the two components. It is known that tracking a moving texture can produce a compelling motion aftereffect opposite to the direction of tracking, even though the tracking tends to stabilize the moving stimulus on the retina (Anstis & Gregory, 1965). Both an extraretinal motion signal (Freeman, Sumnall, & Snowden, 2003) and adaptation to the stationary background (Morgan, Ward, & Brussell, 1976) may be involved. Tracking was not controlled in the experiments of Blaser et al. (2005) and Lankheet and Verstraten (1995), and is thus a possible explanation of their positive findings. However, in a different kind of aftereffect due to attentional tracking, Verstraten, Hooge, Culham, and van Wezel (2001) found no evidence that involuntary pursuit was involved, so we cannot assert that pursuit is a general explanation for adaptation following attentional tracking. Nor did we find an aftereffect of tracking in all of our observers (only in six out of the seven observers in Exp. 6). Future experiments on adaptation to transparent motion, and experiments on “attention” to motion generally, clearly ought to control for pursuit eye movements.

## Electronic supplementary material

Below is the link to the electronic supplementary material.

ESM 1 (DOCX 26 kb)
