# Supplementary material for: Low-level mediation of directionally specific motion aftereffects: Motion perception is not necessary
Source: Atten Percept Psychophys. 2016 Jul 8;78(8):2621–32. doi: 10.3758/s13414-016-1160-1 (PMC5110584; doi:10.3758/s13414-016-1160-1)
Supplement: Supplementary file 1 — (DOCX 26 kb) [file 13414_2016_1160_MOESM1_ESM.docx]

**Supplementary Material**

Demonstration Movies. The mp4 movies available at <https://owncloud.sf.mpg.de/index.php/s/KltJeAUADngWadO>

show representations of the stimuli used in the experiments. These are not identical to the actual stimuli. The frame rate is nominally 50 Hz instead of the 75 Hz used in the experiments, and the colors will depend on the viewing platform. Colors should be adjusted if possible so that red and green dots are equally salient. All the movies were designed to be viewed in a repetitive loop: adapt-probe-probe-adapt-probe-probe-adapt….

**DemoAdaptRedTestRed.mp4** shows the basic 2AFC design used in the experiments. The adapting stimulus (5 s) consists of red dots moving at 30°, with a limited lifetime of 5 frames. This is followed, after a 0.2-s blank interval, by the two probe stimuli (0.5 s each), in sequence. In this case, the first and second probes move with angles of 5° and –5°, respectively. Although they are equally far from the horizontal (0°), the probes should look asymmetrical following adaptation, with the 5° stimulus appearing roughly horizontal and the –5° stimulus shifted clockwise.

**DemoAdaptTransTestRedandGreen.mp4** presents an adaptor consisting of red and green dot streams moving in opposite directions. This is followed by red probes, moving in the same direction as the adapting red dots. As in the previous movie, these probes are moving at +5° and –5°, respectively. The adaptor is then repeated, and followed by green probes. If there were a color-contingent adaptation, the red probes would appear to move in different directions from the green probes.

**DemoAdaptRedTestDVN.mp4** presents a single-direction red adaptor, followed by tests of dynamic visual noise, consisting of stationary but limited-lifetime (5 frame) probes. The probes should appear to drift in the opposite direction to the adaptor.

**DemoAdaptTransTestDVN.mp4** is similar to the previous movie, but the adaptor consists of red and green dots moving in opposite directions. This is followed by two red probes. The adaptor is then repeated and followed by two green probes. The probes may show a drift in the opposite direction to one of the adapting components because of unequal luminance balance, but the question is whether this direction is different for the differently colored probes. Another question is whether the apparent direction of the probes is altered by attending to one of the differently colored adapting components (Lankheet & Verstraten, 1995). Another effect that may be observed is transparent motion in the tests, orthogonal to the adapting axis (Grunewald & Lankheet, 1996).
